# The Field Ration and Feeding of the Territorial Soldier

**Published:** 1910-01-22

**Authors:** 


### The Royal Army Medical Corps Section.

### THE FIELD RATION AND FEEDING OF THE TERRITORIAL SOLDIER.

There has recently been promulgated by the Army Council in connection with the annual training camps of the Territorial Force the very important order that it is not thought necessary, considering the very short time the units are under canvas, to issue dining-tents for their use. The result of this is that such tents can no longer be included in the scales of camp equipment, nor will the Army Council approve of their being provided on the payment of a hiring charge. Before commenting on this memorandum we think this a favourable opportunity to consider both the field ration and the general feeding of the Territorial soldier when called out for active training in the field, for the importance of the relation of the food furnished to the work demanded cannot be over-estimated. At the same time this is not the only standard by which the soldier's food should be judged. It does not suffice that it should supply the daily nutritive wants of the body and furnish the necessary potential energy. There are other requirements to be met, and we think that a dietary to be quite satisfactory should contain a selection and association of different foods that will be both appetising and digestible, that it should receive proper treatment by cooking, and that it should be distributed in appropriate meals at suitable hours and in a comfortable manner. It is by these standards we would judge the Territorial dietary, and it is in reference to these points we propose to consider it. Taking first the two main principles of quantity and quality we think that there can be no complaint on these two points against the Territorial field ration issued by the Government. We regard it as sufficient in amount and excellent of its kind. It consists of one pound of meat and one pound of bread daily with a messing allowance of one shilling a man per diem for the purchase of all necessary supplementary articles as extra bread, butter, milk, cheese, potatoes, vegetables, tea, sugar, etc. The usual plan followed in dealing with this messing allowance is to throw it into a common fund, which is administered by the quartermaster's department of the unit, and out of it all additional articles of food are bought. Last year's experience in camp showed that it is a very fair allowance, so that the Government field ration must be regarded as ample in amount. It was also found to be excellent in quality. Home-fed beef was supplied, but generally New Zealand mutton. The only objection that can be urged against the latter is that it is as a rule too fat, and therefore does not lend itself to successful stewing. In connection with this matter of meat supply we cannot emphasise too much the importance of skilled supervision in connection with it, and as this work in camp may fall to either the sanitary or orderly officer of the day, it is a subject on which all officers should have a practical knowledge, so as to recognise wholesome dead meat, and unfavourable symptoms in live animals that are to serve as food. Of the bread issue all that need be said is that it is not the channel of any special diseases, but as it is the chief source of starch in the soldier's food, care should be taken to see that the loaves are firm, pleasant to the taste, and well baked.

Passing now to the important point of variety in the dietary of the Territorial soldier, it is evident that the Government ration of meat and bread do not furnish much scope for that. Fortunately the daily messing allowance permits of the introduction and selection of different substances that render the meals more appetising and consequently more digestible, and a visit to the kitchens of the various units during the annual camp training would show that advantage has been taken of this fund. It would be found that the regulation issues of meat and bread had been worked up into a series of excellent dishes, mainly through the agency of the articles purchased out of the messing allowance. No special diets are laid down for use in the Territorial camps, but we feel certain that the Territorial soldier who belongs to a big battalion is very well fed while doing duty in the field. He has, as a rule, a breakfast of tea or coffee, with bread and butter, and something in addition, such as fish, fried bacon, or liver, sausages or eggs, and a dinner of meat, roasted or stewed, with potatoes and vegetables, and either soup or pudding as an extra. Lastly, he has an evening meal of tea, with bread and butter, jam or cheese. In smaller units, such as ambulances, the allowance hardly permits of such a generous menu. We cannot give exactly the nutritive value of the above dietary in so many calories, but we feel sure that the Territorial soldier fares as well in many cases during camp training as he does in civil life, and that the food supplied meets all the necessary requirements of the body and furnishes in an agreeable form all the potential energy needed for the discharge of the work demanded from him.

Important as is variety in the soldier's food, equally so is the need for its being properly cooked. No doubt camp cooking has its special difficulties and must be somewhat rough and ready, but what cooking is done should be good and thorough, for by it not only is the food made safer from a health point of view, but it is also rendered more easily masticated and more digestible. Probably stewing and roasting are the methods of cooking best suited for camp, and this seems to be also the view of the military authorities, judging by the cooking apparatus furnished. This consists of one Soyer's stove of 12 gallons capacity for each regiment, and one camp kettle for every eight men, each camp kettle being held sufficient to cook for this number, with vegetables, and for 15 men without. Undoubtedly these camp kettles are admirably adapted for stewing, and tea can be very well made in them, but they are not suitable for roasting. For this reason they do not answer for standing camps, and recourse must be had to one of the many portable stoves that have been devised for military service. Of these there are many excellent ones on the market, but none better than Fowler's stove. Moderate in price, very portable, and easily cleaned, it will cook for 60 to 80 men, and will do all that a range can do in the matter of cooking. It requires for its fire a trench cut in the soil or built up with bricks, but otherwise it is easily worked, and it readily furnishes that variety in cooking that is necessary.

While on the subject of the cooking of food, we sometimes think that the kitchen department of a camp does not always receive the supervision that it ought. We cannot too strongly emphasise the importance of scrupulous care and cleanliness in reference to the utensils used and to the hands and clothes of all the persons who handle them or the food. Facilities for the ablution of the hands should be given the orderly-men of the kitchen, and there should be ample supplies of clean towels for all purposes. Special attention should be paid to the bread and meat stores, so as to keep them tidy, well ventilated, and free from flies, while the kitchen and all its fittings, such as tables, safes, shelves, and utensils should be perfectly clean. The possibility of ptomaine poisoning from imperfectly-cleansed cooking-trays should be borne in mind, and the master-cook should carefully inspect all tins on their return, refusing to accept any not properly cleansed.

As regards the timing of the meals of the Territorial soldier, no exception can be taken to existing arrangements. Theoretically, it might be better to have the evening meal later than the present hour, but as the evening is the only time the Territorial soldier has to himself this would confine him to camp just when he wishes to be free. It is, however, in connection with his messing arrangements or the manner in which his meals will be taken that the order of the Army Council about dining-tents will be most felt. A good deal of controversy has taken place on the question of their employment in camp, and it is not settled yet. Without going into the various arguments for and against them, we are inclined to think that the general consensus of opinion is rather in favour of their use, and this is our view. The tents are the men's sleeping apartments, and as the system of having meals taken there has been now abandoned in barracks, the same principle should hold good in camps. With the dining-marquees the sleeping-tents are kept free of all odds and ends of food, while their use tends to a higher standard of living and of comfort, especially in wet weather. In this way they indirectly increase the health and well-being of the men and also help to make the Territorial service more attractive and popular, as it well deserves to be, for in a week or fortnight of camp training, with its open-air life, its interesting work, and its good food, there lies a store of healthy and inexpensive enjoyment that an ordinary holiday does not always furnish.

